# Effect of Vitamin C Injection on Flexor Tendon Healing in Zone II: A Randomized Controlled Trial

**DOI:** 10.7759/cureus.102075

**Published:** 2026-01-22

**Authors:** Faezeh Tajari, Babak Toloue Ghamari, Meisam Jafari Kafiabadi, Hooman Shariatzade, Farsad Biglari, Omid Mahmoudi Nasab, Nastaran Salavati Mohammadi, Farid Najd Mazhar

**Affiliations:** 1 Physiotherapy Research Center, Shahid Beheshti University of Medical Sciences, Tehran, IRN; 2 School of Medicine, Shohada-e Tajrish Hospital, Shahid Beheshti University of Medical Sciences, Tehran, IRN; 3 Department of Orthopedic Surgery, Shohada-e Tajrish Hospital, Shahid Beheshti University of Medical Sciences, Tehran, IRN; 4 Department of Hand Surgery, Shafa Hospital, Tehran University of Medical Sciences, Tehran, IRN; 5 Bone and Joint Reconstruction Research Center, Iran University of Medical Sciences, Tehran, IRN; 6 Department of Orthopedics, School of Medicine, Iran University of Medical Sciences, Tehran, IRN; 7 Department of Orthopedics and Traumatology, Shafa Yahyaian Hospital, Tehran, IRN

**Keywords:** collagen, flexor tendons, hand injuries, tendon injuries, tendon repair, wound healing

## Abstract

Background

Tendons are connective tissues that link muscles to bones, playing a key role in transmitting force and enabling joint movement. Tendon injuries, such as inflammation and tears, commonly occur due to excessive strain or repetitive motions, causing pain and decreased function. Because of its important role in collagen production and antioxidant effects, vitamin C has been suggested as a supplement to support healing in tendon injuries. This study aims to assess the impact of vitamin C injections on recovery from tendon tears in patients after surgery.

Methods

This study was conducted as a randomized controlled clinical trial. A total of 30 patients scheduled for flexor tendon repair who met the inclusion criteria were randomly assigned to either the intervention group or the control group. The intervention group received vitamin C injections after surgery, while the control group received standard care. Outcomes were assessed using range of motion (ROM) metrics and the Strickland Rating Scale (SRS) to compare the recovery results between the two groups.

Results

The findings showed that the ROM and the SRS scores improved in the intervention group that received vitamin C compared to the control group. However, these differences were not statistically significant (p>0.05). This indicates that while vitamin C might help speed up tendon healing, there was no meaningful difference between the two groups in terms of measurable recovery.

Conclusion

Although preliminary data suggest the potential benefits of vitamin C supplementation in tendon healing, current evidence does not demonstrate an advantage over standard treatment. Further research is necessary to determine its therapeutic role in clinical practice.

## Introduction

Tendon injuries are common and can significantly impact daily activities and physical function. Their incidence varies across populations, with reported rates of approximately 33 per 100,000 person-years [1]. These injuries show characteristic demographic patterns: they tend to occur more frequently in younger adults, particularly those aged 20-29 years, and males are affected more often than females. Most cases involve a single tendon, with extensor tendon injuries reported more often than flexor injuries [2]. These injuries pose considerable challenges in recovery, making effective treatments and innovative strategies essential for improving clinical outcomes [3]. Among the tendons of the human body, the flexor tendons of the hand, particularly those located in Zone II, are highly vulnerable to injury [4]. This anatomical zone is often referred to as "no man's land" due to its intricate structure, comprising tendons, synovial sheaths, and pulleys [5]. Injuries in this region not only impair hand function but also present unique challenges to surgeons, as postoperative adhesions and limited tendon gliding can hinder recovery [6].

The natural healing of flexor tendons involves a complex interplay of inflammation, collagen deposition, and tissue remodeling [7]. However, excessive fibrosis during the healing process can result in adhesions that compromise the tendon's ability to glide within its sheath [8]. Such outcomes can significantly impair the hand's functional recovery and quality of life for patients. As a result, research continues to focus on strategies to enhance tendon healing while minimizing complications such as adhesions and stiffness [6].

Vitamin C, an essential water-soluble vitamin, plays a pivotal role in connective tissue health, particularly through its involvement in collagen synthesis [9]. Collagen is a fundamental structural protein that contributes to the strength and elasticity of tendons [10]. Additionally, vitamin C acts as a cofactor for prolyl and lysyl hydroxylases, enzymes crucial for stabilizing and cross-linking collagen molecules [11]. Beyond collagen synthesis, vitamin C's potent antioxidant properties help mitigate oxidative stress, a key contributor to tissue damage and delayed healing in post-surgical settings [12].

Preclinical studies have demonstrated that vitamin C supplementation can enhance collagen production, reduce inflammation, and accelerate the repair of injured tendons [12]. However, clinical evidence on its direct application in tendon repair remains limited. While oral supplementation has been widely studied, localized injection of vitamin C into the affected tissue offers a targeted approach, potentially delivering higher concentrations of the vitamin to the site of injury and maximizing its therapeutic benefits [13].

The primary aim of this study was to investigate the effects of vitamin C injection on the healing of flexor tendons in Zone II following surgical repair, specifically, to determine whether vitamin C can improve functional outcomes, such as range of motion (ROM) and tendon strength, and reduce complications such as adhesions. By comparing the outcomes of patients treated with vitamin C with those of patients who received standard treatment, this study contributes to the growing body of knowledge on innovative strategies to improve the outcomes of tendon repairs.

This study has not been previously presented as a poster or meeting abstract. The current manuscript represents the first full presentation of these findings.

## Materials and methods

This randomized controlled clinical trial was conducted at Shohada-e Tajrish Hospital, Tehran, Iran, to evaluate the effect of vitamin C injection on tendon healing after flexor tendon repair in Zone II of the hand. The trial followed the Consolidated Standards of Reporting Trials (CONSORT) 2010 guidelines [14] and was registered with the Iranian Registry of Clinical Trials (IRCT) (registration number: IRCT20211201053235N7). Ethical approval was granted by the Research Ethics Committees of the School of Medicine, Shahid Beheshti University of Medical Sciences (approval number: IR.SBMU.MSP.REC.1401.593), and all participants provided written informed consent before enrollment. No modifications were made to the trial protocol once the study had begun. Participants were randomly assigned in a 1:1 ratio to either the intervention group, which received a local vitamin C injection, or the control group, which received standard postoperative care without vitamin C.

Participants

Thirty adult patients (aged 18-60 years) who presented with traumatic flexor tendon injuries in Zone II requiring surgical repair were included. Exclusion criteria comprised previous tendon surgery, systemic or connective tissue disorders (such as diabetes mellitus, rheumatoid arthritis, or peripheral vascular disease), smoking, systemic vitamin supplementation, pregnancy or lactation, psychiatric or cognitive disorders, severe comorbidities, and any known allergy to vitamin C.

Randomization and blinding

Randomization was performed using a block method (block sizes of four and six) with a 1:1 allocation ratio, generated by an independent statistician through computer-based random numbers. Allocation concealment was ensured by sequentially numbered, opaque, sealed envelopes (SNOSE), which were opened only after surgery by a clinician not involved in treatment or outcome assessment. Because of the nature of the intervention, patients and surgeons were not blinded; however, postoperative rehabilitation protocols were strictly standardized across both groups, and outcome assessors measuring ROM and Strickland scores were blinded to group allocation. Data analysts were also kept blinded until the statistical analyses were finalized to reduce detection bias. No interim analyses were conducted.

Surgical technique and intervention

All procedures were performed by the same orthopedic team to maintain consistency. Under regional anesthesia, tendon repair was carried out using a four-strand modified Kessler technique with 3-0 nylon under loupe magnification and reinforced by a running non-locking epitendinous suture with 5-0 nylon. When the digital nerve was injured, it was repaired under magnification to restore alignment and function. Before wound closure, 500 mg of vitamin C (ascorbic acid) diluted in 5 mL of normal saline was injected locally into the peritendinous soft tissues surrounding the repaired flexor tendon, avoiding intratendinous injection. This dose was selected based on its established safety profile, local availability, and prior experimental and clinical evidence suggesting that localized vitamin C exposure may enhance collagen synthesis and reduce adhesion formation. The injection was performed immediately prior to wound closure to allow local tissue exposure during the early inflammatory phase of tendon healing. No placebo or saline injection was administered in the control group.

After closure, a dorsal blocking splint was applied with the wrist in slight flexion and the metacarpophalangeal joints flexed at 70-90°. The splint remained in place for three weeks. Controlled passive flexion and active extension began immediately after surgery, active motion exercises started at six weeks, and full mobilization was encouraged after eight weeks. No major deviations were observed. Patients were monitored for adhesions, re-rupture, and other complications throughout the follow-up period. All patients followed the same standardized rehabilitation protocol, and adherence was reinforced and reviewed at each follow-up visit.

Outcome measures

**Figure 1:**
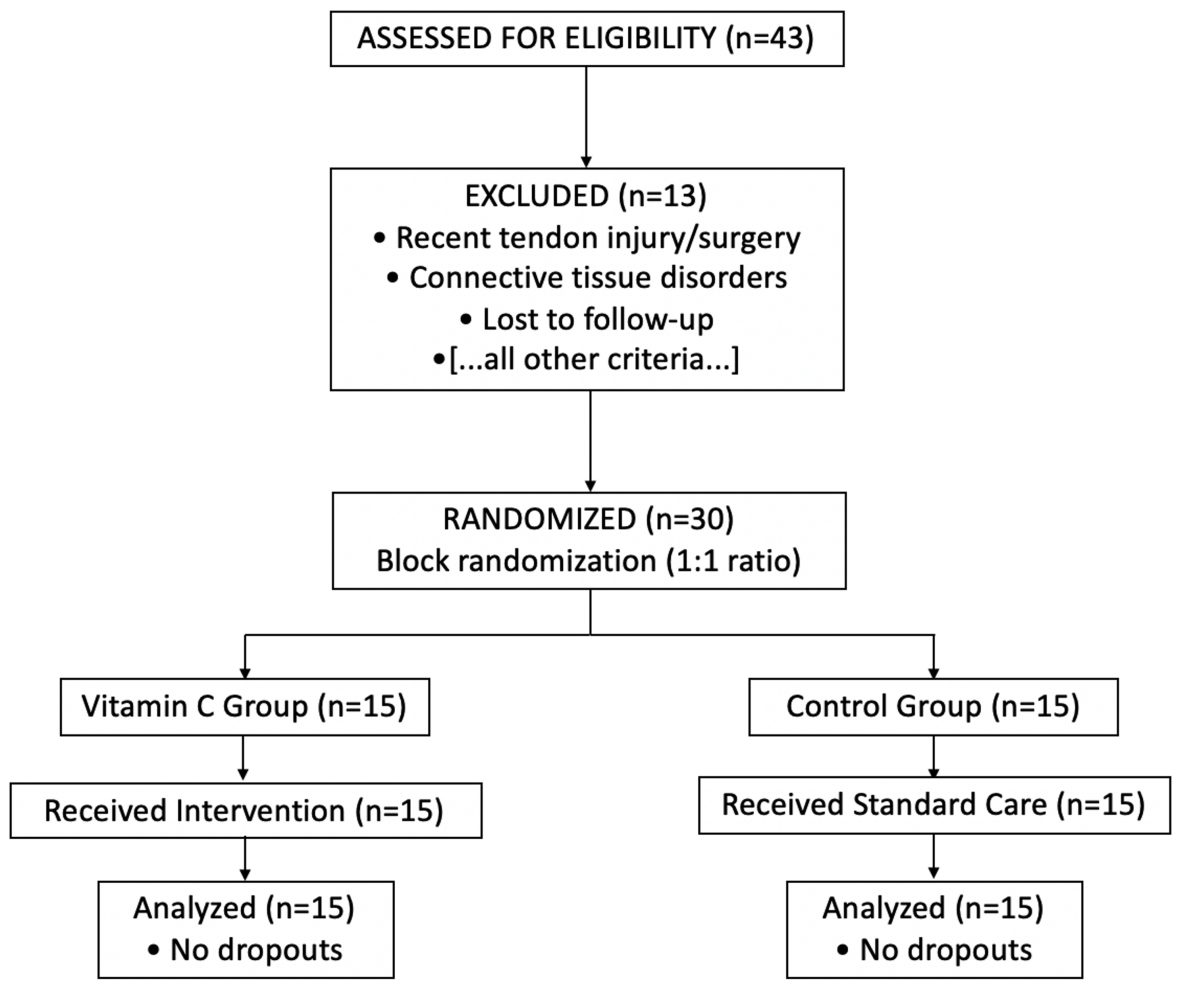
CONSORT flow diagram of participant enrollment, allocation, and analysis CONSORT: Consolidated Standards of Reporting Trials

The primary outcomes were as follows: ROM was assessed as total active motion (TAM), calculated as the sum of active flexion minus extension lag at the metacarpophalangeal, proximal interphalangeal, and distal interphalangeal joints of the affected digit [15]. Functional recovery was evaluated using the Strickland scoring system [16] based on total active flexion and extension deficit. Secondary outcomes included the occurrence of adhesions, tendon rupture, and other postoperative complications. All patient data were collected using a standardized checklist. The flow of participant screening, randomization, allocation, and analysis is illustrated in the CONSORT diagram (Figure 1).

Sample size calculation

An a priori power analysis using G*Power Ver. 3.1 (Heinrich-Heine-Universität Düsseldorf, Düsseldorf, Germany) estimated the required sample size. Assuming a medium effect size (Cohen's d=0.5) for improvement in ROM at 12 weeks, with α=0.05 (two-tailed) and power=0.80, the minimum sample size was 26 participants (13 per group). To allow for possible dropouts and improve statistical reliability, 30 patients (15 per group) were recruited. The observed effect size was smaller than anticipated, which may explain the lack of statistical significance in some comparisons.

Statistical analysis

All analyses were performed with IBM SPSS Statistics for Windows, V. 25.0 (IBM Corp., Armonk, NY, USA). In addition to between-group comparisons at individual time points, longitudinal changes in ROM were analyzed using a linear mixed-effects model with fixed effects for group, time, and group-by-time interaction and a random intercept for subjects to account for within-subject correlation. Relevant test statistics (t or χ²) and corresponding p-values were reported in each table. A p-value of <0.05 was considered statistically significant.

## Results

A total of 30 patients were included, with 15 participants in each group. The average age was 35±10 years in the intervention group and 37±12 years in the control group. The overall male-to-female ratio was 60% (n=9) male and 40% (n=6) female in each group. Baseline characteristics, including mechanism of injury and affected hand, were comparable between groups (p>0.05) (Table 1).

**Table 1:** Baseline characteristics of the study participants Data are shown as mean±SD or n (%). Independent t-test and chi-squared test were used as appropriate. The significance level was set at p<0.05.

Characteristic	Intervention group (n=15)	Control group (n=15)	P-value
Age (years, mean±SD)	35±10	37±12	>0.05
Male participants, n (%)	9 (60%)	9 (60%)	>0.05
Mechanism of injury	Comparable	Comparable	N/A
Affected hand, n (%)	Right: 8 (53%); left: 7 (47%)	Right: 7 (47%); left: 8 (53%)	>0.05

ROM

At four weeks postoperatively, the mean active flexion was 120°±10° in the intervention group and 115°±12° in the control group. By 12 weeks, the mean ROM improved to 140°±8° and 132°±9°, respectively. Although the intervention group consistently showed greater ROM, the difference was not statistically significant (t(28)=1.23; p=0.23) (Table 2).

**Table 2:** Primary outcome: ROM (degrees) ROM was measured using a goniometer at four, eight, and 12 weeks postoperatively. Independent t-tests were used for comparisons between groups. Statistical significance was defined as p<0.05. ROM: range of motion

Time point	Intervention group (mean±SD)	Control group (mean±SD)	t(df)	P-value
Week 4	120°±10°	115°±12°	t(28)=1.23	0.23
Week 8	130°±9°	125°±10°	t(28)=1.35	0.19
Week 12	140°±8°	132°±9°	t(28)=1.46	0.16

Strickland score

At four weeks, the mean Strickland score was 60%±5% in the intervention group and 55%±7% in the control group. By 12 weeks, the scores improved to 75%±6% and 70%±8%, respectively. Despite higher functional recovery trends in the vitamin C group, differences remained statistically non-significant (t(28)=1.46; p=0.16) (Table 3).

**Table 3:** Strickland scores over time Strickland scores were calculated based on active flexion and extension deficit at each time point. Independent t-tests were used for between-group analysis. Statistical significance was defined as p<0.05.

Time point	Intervention group (mean±SD)	Control group (mean±SD)	t(df)	P-value
Week 4	60%±5%	55%±7%	t(28)=1.32	0.20
Week 8	70%±6%	65%±7%	t(28)=1.39	0.18
Week 12	75%±6%	70%±8%	t(28)=1.46	0.16

Across the study population, 26 patients (86.7%) were right-handed and four (13.3%) were left-handed. The dominant hand was injured in 20 patients (66.7%), while 10 patients (33.3%) sustained injury to the non-dominant hand.

In the intervention group, injury causes included knife cuts in nine patients (60%), glass lacerations in five (33.3%), and saw injuries in one (6.7%). In the control group, 11 patients (73.3%) were injured by knives, two (13.3%) by glass, and two (13.3%) by saws. Digital nerve involvement occurred in eight patients (53%) in each group. These data are summarized in Table 4.

**Table 4:** Distribution of injury causes and nerve involvement Chi-squared tests were used to compare categorical variables between groups. Data are presented as n (%). A p-value of <0.05 was considered statistically significant.

Variable	Intervention group (n=15)	Control group (n=15)	P-value
Knife injuries, n (%)	9 (60%)	11 (73.3%)	>0.05
Glass injuries, n (%)	5 (33.3%)	2 (13.3%)	>0.05
Saw injuries, n (%)	1 (6.7%)	2 (13.3%)	>0.05
Digital nerve involvement, n (%)	8 (53%)	8 (53%)	N/A

The distribution of injury mechanism (sharp versus saw injuries) and digital nerve involvement was comparable between the intervention and control groups. Across follow-up visits at four, eight, and 12 weeks, both groups demonstrated progressive improvement in ROM, with consistently higher mean values observed in the vitamin C group at each time point.

Adhesion Formation

Adhesions developed in two patients (13.3%) in the intervention group and three patients (20%) in the control group, showing a lower rate in the vitamin C group, though not statistically significant (χ²(1)=0.24; p=0.62) (Table 5).

**Table 5:** Postoperative complications Data are presented as n (%). The chi-squared test was used for group comparisons. Statistical significance was set at p<0.05.

Complication	Intervention group (n=15)	Control group (n=15)	χ²(df)	P-value
Adhesion formation, n (%)	2 (13.3%)	3 (20%)	χ²(1)=0.24	0.62
Tendon rupture, n (%)	0 (0%)	0 (0%)	N/A	N/A

Tendon Rupture

No tendon ruptures occurred in either group throughout the 12-week follow-up period.

Safety and Tolerability

Vitamin C injections were well tolerated, with no adverse events or allergic reactions observed in any participant.

Overall, the vitamin C-treated group demonstrated slightly better functional outcomes and fewer complications, although these differences were not statistically significant. The consistent improvement trend suggests a potential therapeutic benefit that warrants investigation in larger studies.

## Discussion

Flexor tendon injuries represent one of the most technically demanding challenges in hand surgery, particularly within Zone II, often referred to as "no man's land." This region's complex anatomy, including dense pulley systems and closely associated neurovascular structures, complicates both the surgical repair and the postoperative healing process [4,5]. Despite advances in microsurgical techniques and rehabilitation protocols, tendon adhesion and restricted gliding motion remain significant clinical problems [6,17]. The current study explored the potential role of vitamin C as an adjunct therapy to improve tendon healing outcomes following surgical repair in this critical anatomical zone.

Vitamin C (ascorbic acid) plays an essential role in collagen synthesis by acting as a cofactor for prolyl and lysyl hydroxylase, enzymes required for the hydroxylation of proline and lysine residues that stabilize the collagen triple helix [11]. Collagen constitutes approximately 85-95% of the dry weight of tendons and is the primary determinant of their tensile strength and elasticity [10]. Vitamin C deficiency has long been known to impair collagen formation, resulting in fragile connective tissues and delayed wound healing. Beyond its role as an essential cofactor in collagen synthesis, vitamin C serves as a potent antioxidant, scavenging reactive oxygen species and mitigating oxidative stress that interferes with fibroblast proliferation and extracellular matrix organization [9,12,18,19].

Experimental evidence has confirmed that vitamin C promotes fibroblast differentiation, angiogenesis, and collagen deposition, all of which contribute to improved tendon healing [9,20]. Hung et al. demonstrated that local vitamin C injection reduced adhesion formation and enhanced tendon gliding in a chicken model of flexor digitorum profundus repair [21]. Similarly, Souza et al. reported that vitamin C accelerated collagen maturation and improved biomechanical strength in rat Achilles tendon repairs [22]. These mechanisms support the hypothesis that vitamin C supplementation may improve the microenvironment for tendon regeneration.

In our study, patients treated with local vitamin C injections showed a consistent trend toward improved ROM and higher Strickland scores at all follow-up intervals, although these differences did not reach statistical significance. These findings are in line with previous preclinical research showing that vitamin C enhances tendon remodeling and reduces fibrosis [12,23]. Çelik et al. demonstrated improved collagen organization and higher tensile strength in rats treated with vitamin C compared to untreated controls [24]. Martel et al. also found that vitamin C supplementation enhanced the healing response in rotator cuff injuries by improving collagen cross-linking and angiogenesis [25]. Although most of these data originate from animal models, the biological plausibility and the direction of effect observed in this clinical trial are consistent with those experimental findings.

One potential reason for the lack of statistical significance in our results could be the limited sample size and short follow-up period. Some orthopedic studies suggest that the potential benefits of vitamin C may be modest and may require larger, adequately powered studies with longer follow-up to be detected [25]. Additionally, most animal and in vitro models use repeated or systemic vitamin C administration, whereas our study used a single local injection. The relatively short half-life of ascorbic acid in tissues may limit its duration of effect, suggesting that sustained or repeated delivery might yield stronger clinical outcomes [9,25].

Although the between-group differences did not reach statistical significance and we did not predefine a minimal clinically important difference (MCID) or collect patient-reported outcome measures, the vitamin C group showed a numerically favorable trend in ROM and functional scores across follow-up time points. These findings should be interpreted cautiously and warrant confirmation in larger, adequately powered studies incorporating MCID and patient-reported outcomes. Small gains in early mobility and tendon flexibility can translate into better long-term functional outcomes and reduced stiffness. Importantly, no adverse reactions or allergic responses were observed, confirming that local vitamin C injection is safe and well tolerated.

Our results also reinforce the notion that tendon healing is multifactorial. Mechanical stress, suture technique, vascular supply, and postoperative mobilization all influence the final result [4,26]. While vitamin C can enhance the biological environment through collagen regulation and antioxidant defense, these benefits may be masked by mechanical or surgical factors that exert stronger effects on the outcome. Additionally, although a standardized rehabilitation protocol was used and adherence was reinforced at follow-up visits, adherence was not formally quantified and may have contributed to interindividual variability.

From a clinical standpoint, the simplicity, safety, and low cost of vitamin C make it an attractive adjunctive option. Unlike anti-adhesion agents or mechanical barriers, local vitamin C may provide biochemical support at the repair site without introducing foreign materials or added surgical complexity. This local administration offers a targeted approach and avoids systemic administration. If validated in larger studies, this approach could offer a cost-effective method to improve tendon healing outcomes, especially in resource-limited settings. Furthermore, combining vitamin C with other biological therapies, such as platelet-rich plasma (PRP), stem cell-derived exosomes, or hyaluronic acid, might create synergistic effects that further enhance healing [26,27]. These multimodal strategies align with current trends in regenerative orthopedics, which emphasize augmenting the natural healing response rather than replacing biological tissues.

Study limitations

This study has several important limitations. First, the relatively small sample size (n=30) limits the statistical power to detect differences between groups. Second, the 12-week follow-up period might not have been sufficient to evaluate long-term tendon remodeling or delayed complications such as adhesion recurrence. Third, although randomization and blinded assessment were implemented, the absence of a placebo injection could have introduced procedural bias. Fourth, no biochemical or histological analyses were performed to confirm the molecular effects of vitamin C on collagen synthesis. One limitation of this study is that baseline serum vitamin C levels were not measured. Routine assessment of serum vitamin C is not part of standard clinical care in patients undergoing acute flexor tendon repair and was not feasible within the study setting. However, to reduce potential confounding, patients receiving systemic vitamin C supplementation were excluded, and randomization was expected to distribute baseline nutritional status evenly between groups. Future studies incorporating baseline and longitudinal assessment of serum vitamin C levels may help further clarify the relationship between systemic vitamin C status and tendon healing outcomes. Lastly, this was a single-center study, which may limit the generalizability of the results. Nevertheless, the consistent trend toward improved outcomes and the absence of adverse events support the potential of vitamin C as a safe, biologically plausible adjunct in tendon repair.

Future directions

Future research should focus on optimizing the dosage, timing, and route of vitamin C administration. Sustained-release formulations or repeated local injections could help maintain effective concentrations at the injury site. In addition, combining vitamin C with other biologic agents such as PRP or stem cell-derived exosomes could further enhance regenerative outcomes [26,27]. Future multicenter randomized controlled trials with larger samples and longer follow-up are warranted to validate these findings and to better define the clinical relevance of vitamin C in tendon surgery.

## Conclusions

Local vitamin C injection at the time of Zone II flexor tendon repair was feasible and well tolerated, with no treatment-related adverse events observed. Although the vitamin C group showed numerically favorable trends in ROM, Strickland scores, and adhesion rates, the between-group differences did not reach statistical significance in this pilot trial. Larger, adequately powered randomized studies with longer follow-up and patient-reported outcomes are needed to determine whether local vitamin C provides a clinically meaningful benefit. Although the results did not reach statistical significance, the biological rationale, preclinical support, and favorable safety profile justify further investigation through larger, high-quality clinical trials.
